# Multiple, eruptive epitheloide Hämangiome der Kopfhaut

**DOI:** 10.1007/s00105-023-05193-8

**Published:** 2023-07-26

**Authors:** Silvia Mihalceanu, Sarah Schäfer, Thomas Mentzel, Ferdinand Toberer

**Affiliations:** 1 Universitäts-Hautklinik Heidelberg, Ruprecht-Karls Universität Heidelberg, Heidelberg, Deutschland; 2 MVZ Dermatopathologie Friedrichshafen/Bodensee, Friedrichshafen, Deutschland

**Keywords:** Epitheloides Hämangiom, Angiolymphoide Hyperplasie mit Eosinophilie, Benigne vaskuläre Neoplasie, EH, ALHE, Epitheloid hemangioma, Angiolymphoid hyperplasia with eosinophilia, Benign vascular neoplasm, EH, ALHE

## Abstract

Das epitheloide Hämangiom ist eine benigne vaskuläre Neoplasie mit einem charakteristischen histologischen und immunhistochemischen Muster, insbesondere gekennzeichnet durch ein lymphozytäres Entzündungsinfiltrat mit beigemengten Eosinophilen und eine FOS-B-Expression. Die Abklärung der Diagnose ist von besonderem Stellenwert, da differenzialdiagnostisch auch maligne epitheloidzellig differenzierte vaskuläre Tumoren infrage kommen. Wir präsentieren eine Patientin mit multiplen epitheloiden Hämangiomen der Kopfhaut, begleitet von starken Schmerzen und Juckreiz. Die lange Vorgeschichte mit multiplen Therapieversuchen verdeutlicht den oft begrenzten Erfolg der aktuell zur Verfügung stehenden Behandlungsmodalitäten.

## Abstract (en)

Epithelioid hemangioma is a benign vascular neoplasm with a characteristic histological and immunohistochemical pattern, characterized by a lymphocytic inflammatory infiltrate with admixed eosinophils and FOS‑B expression. The correct diagnosis is of particular relevance, since malignant vascular tumors with differentiated epithelioid cells can also be considered in the differential diagnosis. We present a patient with multiple epithelioid hemangiomas of the scalp accompanied by severe pain and itching. The long history of multiple therapeutic attempts illustrates the limited success of currently available treatment options.

## Anamnese

Eine 55-jährige Patientin stellte sich im September 2022 mit multiplen, schmerzhaften und juckenden Knoten an der Kopfhaut in unserer Ambulanz vor.

Die Symptomatik begann im Sommer 2018 mit Juckreiz im Bereich der Kopfhaut. Im Herbst 2018 trat dann retroaurikulär rechts ein solitärer Knoten auf, eine Lokaltherapie mit einem Kombinationspräparat aus Prednisolon 2 mg/ml und Salicylsäure 4 mg/l (3-mal/Tag für insgesamt 8 Wochen) und mit 10 % Macrogollaurylether in Basiscreme DAC (Deutsches Arzneimittel-Codex) (1-mal/Tag für 4 Wochen) brachte keine Linderung. Bis April 2019 kamen ca. 10 weitere, nun auch schmerzhafte Nodi hinzu. Ablative Therapieversuche mittels Kryotherapie, PDL (Pulsed-Dye-Laser, 595 nm, 10-mm-Spotgröße, 7 J/cm^2^, 0,45 ms Pulsdauer und dynamischer Kühlung), CO_2_-Laser (Kohlenstoffdioxidlaser, 10.600 nm, 6 J/cm^2^, Pulsdauer = 10 ms, wiederholt in 0,1-s-Intervallen) sowie die chirurgische Exzision mehrerer Knoten zeigten sich wenig erfolgreich, da diese ein rezidivierendes Wachstumsmuster aufwiesen. Im Herbst 2021 erfolgte probatorisch auch eine intraläsionale Glukokortikoidinjektion mit Triamcinolonacetonid mehrerer Knoten, die jedoch sine effectu war.

Insbesondere der Schmerz im Bereich der Knoten wurde als sehr stark beschrieben, sowohl auf Druck als auch in Ruhe, mit einer Intensität bis zu 8/10 auf der visuellen Analogskala, weshalb die Patientin zum Zeitpunkt der Vorstellung eine analgetische Bedarfsmedikation nach WHO-Stufe II mit Metamizol, Diclofenac und Tilidin hatte. Ein Therapieversuch mit Pregabalin 150 mg 2‑mal/Tag führte zu keiner Schmerzreduktion. Zusätzlich sei eine Eigenbehandlung mit dem Phytocanabinoid Canabidiol (CBD-Öl) in einer Dosierung von bis zu 35 % erfolgt, welche anamnestisch eine Linderung der Symptomatik erzielen konnte.

Bekannte Nebenerkrankungen waren ein Zustand nach totaler Hysterektomie mit Adnexektomie bei Uterus myomatosus und Endometriumkarzinom FIGO (Fédération Internationale de Gynécologie et d’Obstétrique) Stadium I. Eine Dauermedikation jenseits der Analgetikaeinnahme bestand nicht.

## Klinischer Befund

**Figure 1:**
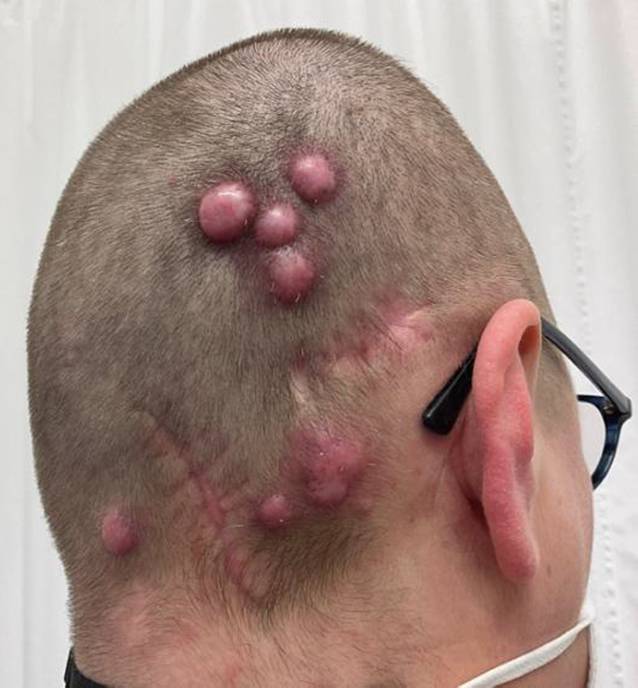

Die dermatologische Inspektion des Capillitiums zeigte parietookzipital rechts, retroaurikulär rechts und nuchal rechts multiple, pralle, derbe und gut verschiebliche erythematöse Nodi mit Teleangiektasien (Abb. 1).

Die Läsionen imponierten gruppiert in dem jeweiligen Areal, das restliche Integument war von ähnlichen Hautveränderungen frei.

## Histologie und weitere Diagnostik

Es wurden in 3 Sitzungen insgesamt 9 Knoten okzipital, nuchal und retroaurikulär rechts bis in das subkutane Fettgewebe exzidiert. Alle Exzisate wiesen eine identische Histologie auf.

**Figure 2:**
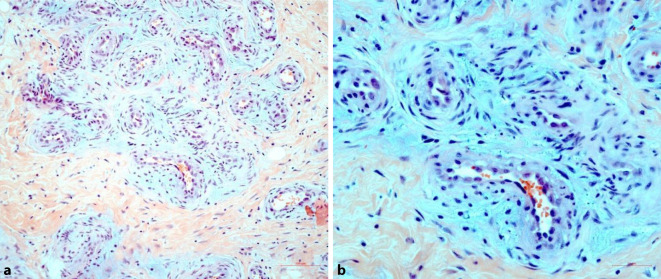

Unter einem mittelbreiten, regelrecht stratifizierten Epithel zeigte sich jeweils ein dermal-subkutan gelegener Gefäßtumor, der relativ gut umschrieben war und einen organoiden Aufbau aufwies. Es zeigten sich Gefäße mit unterschiedlich großen Lumina und teils starken Wandkonfigurationen. Die Endothelien wiesen dabei eine epitheloide Morphologie auf, wobei die Lumina teils durch die polsterartig vorgewölbten Endothelien etwas eingeengt erschienen (Abb. 2b). Höhergradige zytologische Atypien im Bereich der Endothelien fanden sich nicht. Begleitend zeigte sich ein lymphozytäres Entzündungsinfiltrat mit beigemengten eosinophilen Granulozyten (Abb. 2a). Fokal zeigten sich zudem regelrechte kleine Lymphfollikel im Bereich des Tumors und seiner Umgebung.

**Figure 3:**
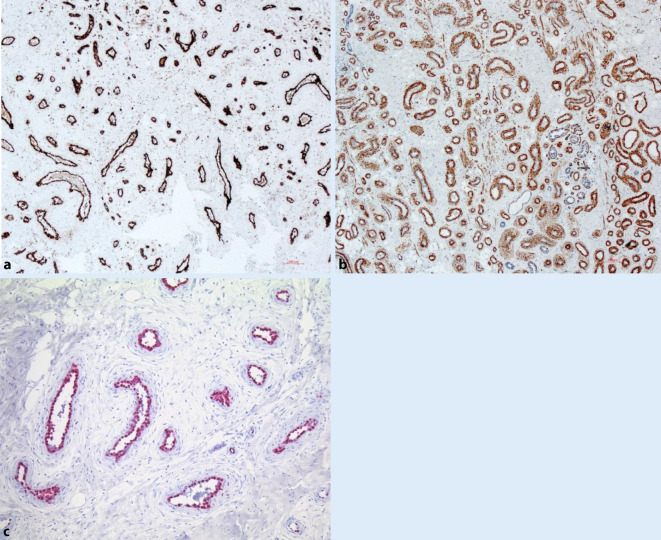

Immunhistochemisch zeigte sich eine homogene Darstellung der Gefäßendothelien in der CD31-Färbung (Abb. 3a). In der Alpha-SMA(„Smooth Muscle Actin“)-Färbung zeigte sich eine erhaltene Perizytenmanschette (Abb. 3b). In der Ki67-Färbung zeigten sich lediglich vereinzelte Endothelzellen positiv gefärbt. Die Tumoren waren jeweils positiv für FOS‑B (v-fos FBJ murine osteosarcoma viral oncogene homolog) (Abb. 3c; Tab. 1).

**Table 1:** 

Antikörper	Klon	Herkunft	Hersteller	Verdünnung	Antigen Gewinnung
CD31	JC70	Maus	Cell Marque, Rocklin, CA, USA	RTU	Ultra CC1, Roche
Ki67	30‑9	Hase	Roche, Basel, Schweiz	RTU	Ultra CC1, Roche
ASMA	1A4	Maus	Cell Marque, Rocklin, CA, USA	RTU	Ultra CC1, Roche
FOS‑B	5G4	Hase	Cell Signaling, Danver, MA, USA	1:100	Vorbehandlung bei pH 6,0 ohne Enzym

*RTU* „ready to use“

Ein extern durchgeführtes MRT des Kopfes zeigte keine intrakraniellen Auffälligkeiten und sichtbare subkutan gelegene Hämangiome mit erhöhter homogener Signalintensität auf T2-gewichteten Sequenzen.

## Diagnose

Bei der Patientin wurden multiple, eruptive epitheloide Hämangiome diagnostiziert.

## Therapie und Verlauf

Bei der Patientin entschieden wir uns für die sequenzielle histographisch kontrollierte Exzision der Tumoren im ambulanten Setting sowie eine engmaschige klinische Kontrolle. In einem Zeitraum von 6 Monaten erfolgte bislang die mikrographisch kontrollierte Exzision von 6 Nodi. Die Patientin zeigte sich soweit rezidivfrei, wir planen jedoch ein engmaschiges Follow-up alle 3 bis 6 Monate für die nächsten 5 Jahre.

## Diskussion

Das epitheloide Hämangiom (EH, Synonym: angiolymphoide Hyperplasie mit Eosinophilie [ALHE]) ist eine benigne lymphoproliferative Neoplasie aus der Familie der epitheloidzellig differenzierten vaskulären Tumoren und wurde erstmals 1969 von Wells und Whimster beschrieben [1]. Die Erkrankung manifestiert sich vorwiegend zwischen der 2. und 5. Lebensdekade, kann jedoch in jedem Alter vorkommen. Männer und Frauen sind gleichermaßen betroffen, die häufigsten Lokalisationen sind Kopf und Hals, beobachtet werden jedoch auch Läsionen am Rumpf, an den Extremitäten und im Genitalbereich [2]. Extrakutane Manifestationen sind selten und wurden im Bereich der Mundschleimhaut, der Orbita, der Parotis, des Dickdarms oder der Knochen beschrieben. Klinisch imponieren die epitheloiden Hämangiome als multizentrische, dermal oder subkutan gelegene, rot-braune Papeln und Knoten. Die ALHE kann asymptomatisch sein oder Symptome wie Juckreiz, Schmerzen oder spontane Blutungen verursachen. Histologisch ist das epitheloide Hämangiom durch eine vaskuläre Proliferation mit polsternagelartig protuberierenden, zytoplasmareichen epitheloiden Endothelzellen mit umgebendem lymphozytärem und eosinophilem Infiltrat gekennzeichnet [3].

Die Ätiopathogenese dieser eruptiven vaskulären Läsionen ist umstritten. Gegenwärtig werden epitheloide Hämangiome als gutartige vaskuläre Hyperplasien betrachtet, diskutiert werden Zusammenhänge mit arteriovenösen Fisteln und vaskulären Malformationen [4]. Reaktive Proliferationen nach Traumata, hormonelle Veränderungen und Infektionen mit dem humanen T‑lymphotropen Virus (HTLV) oder mit dem humanen Herpesvirus 8 (HHV-8) könnten der Literatur zufolge auch eine Rolle bei der Pathogenese spielen. Beschrieben wurden auch Assoziationen von ALHE mit nephrotischem Syndrom und Schwangerschaft. In einigen Fällen fanden die Autoren eine erhöhte Expression von Interleukin 5 und vaskulärem endothelialem Wachstumsfaktor. Auf genetischer Ebene wurden rezidivierende FOS‑B und GATA3-Genfusionen als ein Schlüsselereignis bei der Entstehung insbesondere der extrakutanen epitheloiden Hämangiome beschrieben [5, 6]. Maurus et al. legten zudem nahe, dass kutane EH anders als extrakutane EH somatische Mutationen in Genen des mitogen-aktivierten Proteinkinasewegs (MAPK) tragen und es sich dabei eher nicht um eine reaktive Neoplasie handelt [7].

Kempf et al. konnten klonal neu angeordnete T‑Zell-Rezeptor(TCR)-Gene nachweisen und legten nahe, dass eine Untergruppe von ALHE-Fällen einen CD4-T-Zell-Ursprung haben und eine lymphoproliferative T‑Zell-Erkrankung benigner oder niedriggradig maligner Natur darstellen könnte [8].

Differenzialdiagnostisch unterscheidet man zum einen weitere epitheloidzellig differenzierte vaskuläre Tumoren wie den benignen kutanen epitheloiden angiomatösen Knoten (CEAN) und maligne Tumoren wie das pseudomyogene Hämangioendotheliom (PM-HAE, „low-grade“), das epitheloide Hämangioendotheliom („low-grade“ oder „high-grade“) und das epitheloide Angiosarkom [9]. Für die immunhistochemische Charakterisierung von epitheloiden Gefäßtumoren schlagen Llamas-Velasco et al. ein Panel von Antikörpern vor, darunter FOS‑B, CAMTA‑1, TFE‑3, AE1/3 und c‑MYC. Dabei zeigt das epitheloide Hämangiom eine Expression von FOS‑B bei gleichzeitiger Negativität für alle anderen aufgeführten immunhistochemischen Marker und wird von den Autoren in eine klassische, eine zelluläre sowie eine inflammatorische Variante (ALHE) unterteilt [10]. Die Nomenklatur ist ebenfalls umstritten, die WHO klassifiziert das EH als benignen vaskulären Weichteiltumor ohne weitere Unterteilung anhand histologischer Merkmale [11].

Zudem gibt es ein breites Spektrum an anderen multifokalen Gefäßproliferationen, die in eruptiver Form auftreten können: glomeruloides Hämangiom, kaposiformes Hämangioendotheliom, büschelartiges Angiom, Angiosarkom, mikrovenuläres Hämangiom, Spindelzellhämangiom und eruptives pyogenes Granulom. Nicht zuletzt sollte bei entsprechender Anamnese auch an infektiöse Gefäßproliferationen wie das Kaposi-Sarkom und die bazilläre Angiomatose gedacht werden [10]. Auch sollte die ALHE von der Kimura-Krankheit unterschieden werden. Diese manifestiert sich mit solitären oder multiplen, subkutanen Knötchen lokalisiert in der periaurikulären und submandibulären Region und betrifft insbesondere junge Männer asiatischer Herkunft. Charakteristisch sind eine regionale Lymphadenopathie mit Eosinophilie und erhöhter Serum-IgE-Spiegel, histologisch zeigen sich prominente Lymphfollikel und Fibrose mit entzündlichem, eosinophilenreichem Infiltrat und proliferierenden postkapillären Venolen [12].

Die therapeutischen Optionen der ALHE umfassen neben der chirurgischen Exzision auch ablative, systemische, intraläsionale und topische Behandlungsmodalitäten, welche insgesamt nur einen begrenzten Erfolg mit hohen Rezidivraten zeigen. Die chirurgische Exzision, insbesondere die mikrographisch kontrollierte Exzision (Mohs-Chirurgie), gilt derzeit als die standardmäßige Therapie und weist hohe Rezidivraten von 33–50 % auf [13]. Daten zu anderen Modalitäten der mikrographisch kontrollierten Exzision haben wir nach unserer Literaturrecherche nicht gefunden. Diese hohen Rezidivraten werden von manchen Autoren auf das aggressive Wachstumsmuster dieser stark vaskularisierten Neoplasie und auf die damit verbundenen Schwierigkeiten, selbst mit mikrographisch kontrollierten Exzisionsmethoden saubere Resektionsränder zu erzielen, zurückgeführt [14].

Andere therapeutische Modalitäten umfassen Laserverfahren (PDL-, Nd:YAG- und CO_2_-Laser) [15], Kryotherapie, photodynamische Therapie und intraläsionale Steroidinjektionen. Bislang eingesetzte systemische Therapien, die auf das vaskuläre proliferative Element der ALHE abzielen, sind Propranolol [16] und Isotretinoin [17]. Letzteres bewirkt aufgrund der antiangiogenen Eigenschaften eine Verringerung der Produktion des vaskulären endothelialen Wachstumsfaktors (VEGF) durch Keratinozyten. Neuere Therapieansätze zielen auf Zytokine (topisches Imiquimod) [18], Interleukin‑5 (Mepolizumab i.v.) [19], IL-4/13 (Dupilumab s.c.) [20] und den vaskulären endothelialen Wachstumsfaktor (Bevacizumab intraläsional) ab [21]. All diese Therapieansätze wurden bislang nur in Fallberichten beschrieben.

Insgesamt verdeutlicht die dargestellte Kasuistik die komplexe diagnostische Abklärung und Behandlung einer Patientin mit multiplen epitheloiden Hämangiomen am Capillitium. Die diagnostische Abklärung und individuelle Therapieentscheidung sind bei dieser seltenen Erkrankung von besonderer Relevanz. Es ist von Interesse, die vorhandenen therapeutischen Optionen im Hinblick auf Effektivität und Rezidivfreiheit weiter zu evaluieren.

## Fazit für die Praxis

- Das epitheloide Hämangiom ist ein seltener benigner Gefäßtumor und manifestiert sich mit gruppierten Nodi und Plaques. Prädilektionsstellen sind die Hals-Kopf-Region, tiefe Varianten und Knochenbefall kommen vor.
- Histopathologische Kennzeichen sind protuberierende zytoplasmareiche epitheloide Endothelien mit eosinophilenreichem lymphozytärem Begleitinfiltrat und fehlenden endothelialen Kernatypien.
- Therapeutische Optionen des EH ermöglichen nur einen begrenzten Erfolg mit hohen Rezidivraten und sollten individuell und ggf. interdisziplinär festgelegt werden.
